# Visual adaptation dominates bimodal visual-motor action adaptation

**DOI:** 10.1038/srep23829

**Published:** 2016-03-31

**Authors:** Stephan de la Rosa, Ylva Ferstl, Heinrich H. Bülthoff

**Affiliations:** 1 Max Planck Institute for Biological Cybernetics, Department of Human Perception, Cognition, and Action, Spemannstr 38, 72076 Tübingen, Germany

## Abstract

A long standing debate revolves around the question whether visual action recognition primarily relies on visual or motor action information. Previous studies mainly examined the contribution of either visual or motor information to action recognition. Yet, the interaction of visual and motor action information is particularly important for understanding action recognition in social interactions, where humans often observe and execute actions at the same time. Here, we behaviourally examined the interaction of visual and motor action recognition processes when participants simultaneously observe and execute actions. We took advantage of behavioural action adaptation effects to investigate behavioural correlates of neural action recognition mechanisms. In line with previous results, we find that prolonged visual exposure (visual adaptation) and prolonged execution of the same action with closed eyes (non-visual motor adaptation) influence action recognition. However, when participants simultaneously adapted visually and motorically – akin to simultaneous execution and observation of actions in social interactions - adaptation effects were only modulated by visual but not motor adaptation. Action recognition, therefore, relies primarily on vision-based action recognition mechanisms in situations that require simultaneous action observation and execution, such as social interactions. The results suggest caution when associating social behaviour in social interactions with motor based information.

How do humans visually recognize the actions of others? Two physiologically plausible accounts have been proposed to explain visual action recognition. The first proposes that action recognition processes are vision-based and mainly rely on the analysis of visual action information^1^,^2^,^3^,^4^,^5^,^6^. The second account highlights the primacy of the motor system in action recognition. Specifically, it suggests that actions are understood by mapping visual action representations onto motor representations of the same action (direct matching)^7^,^8^,^9^,^10^. A central debate in cognitive neuroscience concerns how these two mechanisms contribute to action recognition^11^,^12^,^13^,^14^,^15^. Much of the evidence for either account has been demonstrated by showing that either visual or motor action information alone influence action recognition^16^,^17^,^18^,^19^,^20^. This is in contrast to action recognition in social interactions, where humans simultaneously execute and observe actions. To better understand the use of visual and motor information in action recognition, we examined the interaction of visual and motor action recognition processes in situations of simultaneous action execution and observation.

We investigated the interaction of vision and motor-based action recognition processes using a behavioural adaptation paradigm. In an adaptation paradigm participants receive the same action information, e.g., the visual presentation of a person waving, repeatedly for a prolonged amount of time (adaptation) and report their visual percept of a subsequently presented ambiguous test action. It has been shown that visually adapting to a waving action causes participants to perceive an ambiguous test action, that contains visual elements of both a waving and punching action, more likely as a punch^21^ (adaptation effect).

Adaptation is a useful method to examine behavioural correlates of neural mechanisms^22^,^23^,^24^. Behavioural adaptation effects have been explained in terms of a neural response change induced by the prolonged presentation of the adaptor stimulus. Given that the same neural mechanisms are involved in the processing of the adaptor and the subsequently presented test stimulus, this response change will transfer to the processing of the test stimulus and thereby alter the percept of the test stimulus^24^,^25^. By systematically varying the physical properties between adaptor and test stimulus, one can investigate the tuning properties of the underlying recognition mechanisms. Qualitative comparisons of behavioural action adaptation effects with physiological responses of action sensitive neurons have demonstrated good agreement^26^,^27^,^28^.

We examined the properties of neural mechanisms involved in the classification of social actions, i.e. actions whose action outcome is directed towards another person, by probing humans’ action categorization performance within an adaptation paradigm. Specifically, we examined the visual categorization of social actions in an experimental setup that mimics realistic social interactions by using augmented reality in which three-dimensional avatars were facing participants (Fig. 1A). Participants adapted to a ‘fist bump’ or ‘punch’ action in separate conditions and subsequently categorized an ambiguous action as either a ‘punch’ or ‘fist bump’ (test) (Fig. 1B–D). This dynamic ambiguous test was a video of a morphed action that displayed a weighted linear average of limb joint angles of a punch and a fist bump action.

First, we established the ability of the behavioural adaptation paradigm to measure the effect of both vision and motor-based action recognition processes on visual action recognition. We reasoned that if visual action recognition relies on visual information, visual adaptation to an action should influence the subsequent visual percept of an action. Moreover, motor based accounts propose that motor-visual neural units (e.g. mirror-neurons) are involved in action recognition. According to this suggestion, motor adaptation should alter the response behaviour of these units and thereby change the visual percept of a subsequently perceived action. In two separate unimodal adaptation conditions, we investigated the effect of visual and motor action adaptation on visual action recognition. In the unimodal visual adaptation condition, participants were visually adapted to an action by repeatedly showing them the same adaptor action (‘punch’ or a ‘fist bump’ action). In the unimodal motor adaptation condition participants were motorically adapted to a ‘punch’ or a ‘fist bump’ action by repeatedly executing the adaptor action without visual feedback. In both unimodal adaptation conditions, participants subsequently categorized a visually presented ambiguous action as either ‘punch’ or ‘fist bump’. We subtracted the proportion of ‘punch’ responses in each adaptor condition from proportion of ‘punch’ responses of a baseline condition. In this baseline condition we measured the visual categorization of test actions without prior presentation of an adaptor. The difference in the amount of punch responses between adaptor and baseline condition was used to assess the effectiveness of each adaptor in altering the percept of the ambiguous test stimulus.

## Results

**Figure 2:**
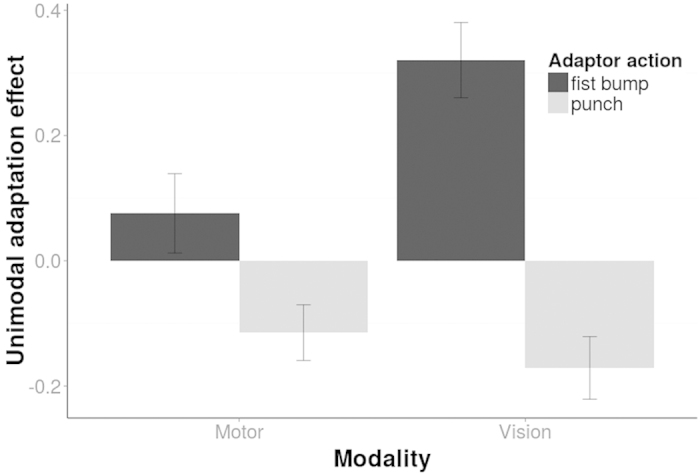
Unimodal adaptation effects shown for the motor (left) and visual (right) modality and each adaptor action (different hues) separately. The unimodal adaptation effect (y-axis) is measured as the difference of proportion ‘punch’ responses between the action adaptation and a no-adaptation (baseline) condition. Both unimodal conditions show an adaptation effect: participants reported the ambiguous test stimuli to look more like a ‘punch’ after being adapted to a fist bump (dark bars) and vice versa. Bars indicate 1 SE of the mean.

We found that both adaptors change the perception of the test stimulus in an antagonistic way. That is, participants were more likely to perceive the test stimulus as a punch after having been adapted to a ‘fist bump’ action and vice versa. An ANOVA examined the influence of the adaptors (fist bump, punch) and modality (vision, motor) on categorization of the test stimulus. We found a significant interaction between modality and adaptors, F(1, 21) = 20.52, p < 0.001. This interaction seems to be owed to motor adaptors inducing a smaller adaptation effect than visual adaptors (Fig. 2). Indeed, the unimodal motor adaptation effect was significantly smaller than the corresponding unimodal visual adaptation effect, t(21) = 4.53, d = 1.09, p < 0.001 (Fig. 2). Yet, both visual and motor action information affect visual action recognition as indicated by a significant unimodal visual, t(21) = 8.09, d = 1.73, p < 0.001, and an unimodal motor adaptation effect, t(21) =  3.32, d = 0.71, p = 0.003 (unimodal adaptation = fist-bump-punch adaptation effect). The behavioural adaptation paradigm is therefore able to measure the effect of motor and vision-based recognition processes on visual action recognition. Moreover, the results indicate that social action categorization is influenced by motor adaptation supporting the idea that the motor system affects action recognition when participants merely observe an action.

**Figure 3:**
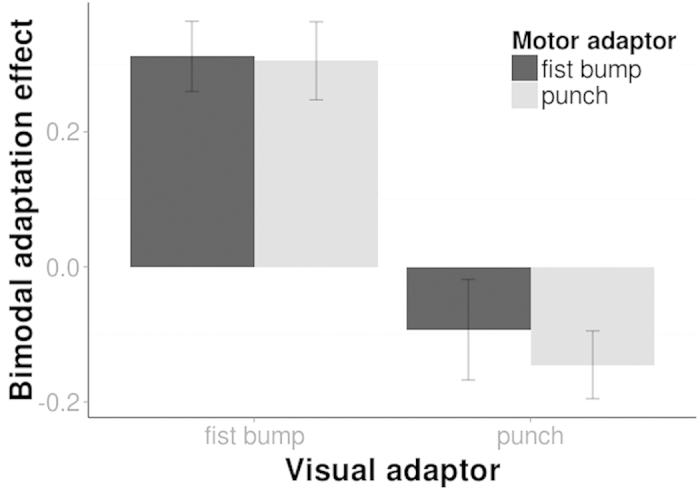
The contribution of the visual action information (along the x axis) and motor action information (different shadings) on bimodal adaptation. Bimodal adaptation effects measured the difference of proportion ‘punch’ responses between the bimodal action adaptation and the no adaptation (baseline) condition. Bimodal adaptation effects were mainly modulated by the visual action information (i.e. along the x-axis) and not motor action information (i.e. different shadings). Bars indicate 1 SE of the mean.

We then explored the interaction between motor and visual action recognition processes by examining the effect of concurrent motor and visual adaptation in bimodal adaptation conditions. In particular, participants adapted simultaneously visually and motorically to either the same or different actions in four conditions (same: punch_vis_/punch_mot_, fist bump_vis_/fist bump_mot_, different: punch_vis_/fist bump_mot_, fist bump_vis_/punch_mot_). For example, during bimodal adaptation to the same action, participants repeatedly executed and observed a punch movement at the same time. Alternatively, during bimodal adaptation to different actions, participants repeatedly executed a punch while they observed a fist bump, and vice versa. In all bimodal adaptation conditions, we probed visual action categorization using the same ambiguous test stimuli as described above. We examined the influence of visual (punch_vis_ vs. fist bump_vis_) and motor (punch_mot_ vs. fist bump_mot_) adaptors on bimodal adaptation effects using a repeated measures ANOVA (Fig. 3). We only found a significant effect of visual adaptation on bimodal adaptation effects, F(1, 21) = 139.06, p < 0.001 suggesting that visual action information drives bimodal adaptation effects (for all other factors p > 0.05).

If unimodal visual adaptation effects dominate bimodal adaptation effects, we expect these two adaptation effects to be significantly related and non-significantly different from each other. Moreover, the dominance of bimodal adaptation effects by unimodal visual adaptation predicts that unimodal motor adaptation should be significantly different and only marginally related to bimodal adaptation effects. We tested these hypotheses by comparing the size and the strength of the relationship between unimodal and bimodal adaptation effects. As for the size of the adaptation effect, a direct comparison of unimodal visual adaptation effects and bimodal adaptation effects, which were averaged to reflect only the visual adaptation manipulation, showed no significant difference, t_paired_(21) = 1.36, p = 0.188. In contrast, bimodal adaptation effects, which were averaged to reflect only the motor adaptation manipulation, were significantly smaller than unimodal motor adaptation effects, t_paired_(21) = 2.49, p = 0.02. Motor action information but not visual action information, therefore, had a significantly reduced effect in bimodal adaptation conditions compared to unimodal adaptation conditions.

**Figure 4:**
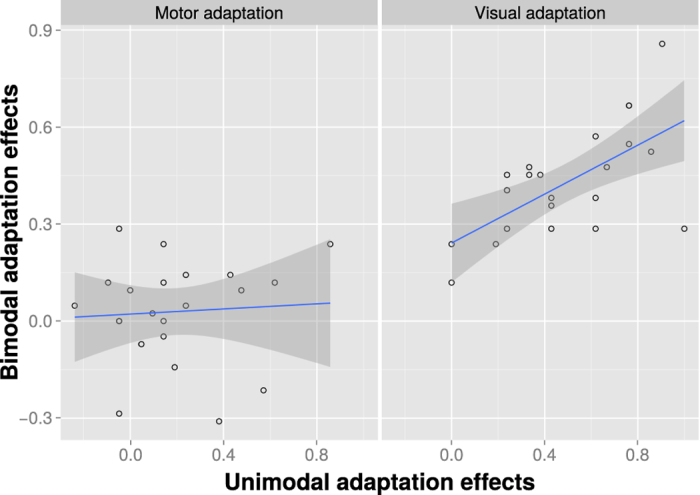
Correlation between unimodal and bimodal adaptation effects. The correlation is shown for the motor (left) and visual (right) adaptation conditions separately. For the calculation of the correlation data was collapsed across the conditions that were not of interest (e.g. collapsing across motor conditions for the calculation of the visual adaptation effect). The gray shaded area represents the 95% confidence band for the parameter estimates.

To examine the relationship between unimodal and bimodal adaptation effects, we correlated unimodal and bimodal adaptation effects. Akin to the analysis of the adaptation effect size, bimodal adaptation effects were averaged to reflect only the manipulation in the modality of interest. The results indicated a significant correlation between unimodal visual adaptation and bimodal adaptation effects, t(20) = 3.66, r = 0.634, p = 0.002, but no significant correlation between unimodal motor adaptation and bimodal adaptation effects, t(20) = 0.30, r = 0.07, p = 0.768 (Fig. 4). A direct comparison of these two correlations revealed a significant difference, t(22) = 2.86, p = 0.01, suggesting that bimodal adaptation effects were more closely related to unimodal visual adaptation effects than to unimodal motor adaptation effects. Overall, these results provide strong support for visual adaptation dominating bimodal adaptation.

**Figure 5:**
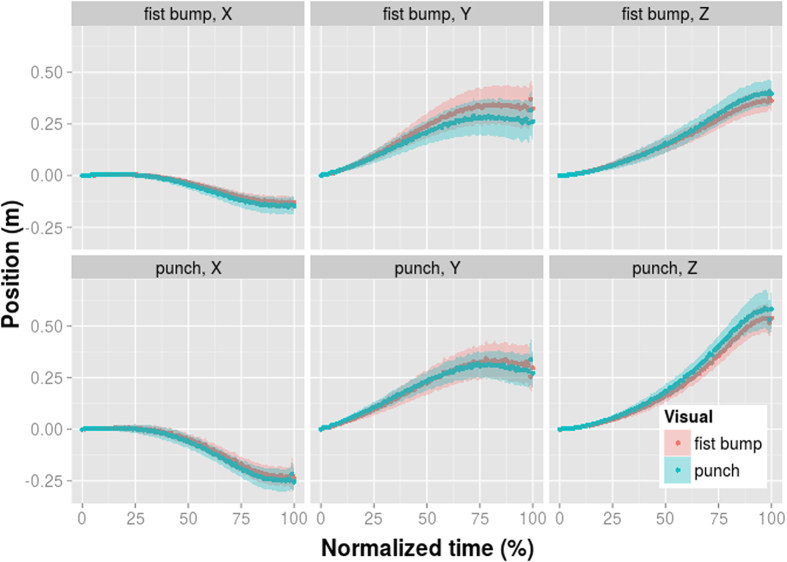
Average spatial position of the hand in the bimodal adaptation control experiment. The motor adaptor conditions are along rows (top row: fist-bump; bottom row: punch) and the visual adaptor conditions in different colours within each panel (red: fist-bump; blue: punch). The hand position along the x (left-right),y (up-down), and z (forwad-backward) axis is shown across different columns. The normalized time expresses each time point as percent of the overall movement time. The shaded area indicates the 95% confidence interval.

A control experiment assessed whether bimodal adaptation effects merely reflect a visual-motor interference effect that caused participants to adjust their motor movement to resemble the visually displayed action. For example, because movement speed is one critical difference between punch and fist bump actions, it was possible that participants might have slowed down or sped-up their hand movement during the concurrent visual presentation of the fist bump or the punch action, respectively. If this were the case, then the motor modality would provide the same action information as the visual modality and, consequently, visual action information were to only seemingly dominate the bimodal adaptation effect. Hence, we repeated the experiment with the bimodal conditions, while we recorded participants’ hand movement speeds. An ANOVA assessing the dependence of hand movement velocity on motor and visual adaptation only showed a significant main effect of motor adaptation, F(1, 14) = 49.05, p < 0.001. No effect involving visual adaptation was significant, p > 0.05. Visual adaptation therefore did not influence participants’ hand speed. Moreover, the overlapping 95% confidence intervals of the hand trajectories between the visual punch and fist-bump adaptor conditions suggests that hand trajectories were not significantly modulated by visual adaptors (Fig. 5).

To ensure that the non-significant effect of visual adaptation on movement speed was not owed to a general ineffectiveness of the visual adaptor in the control experiment, we assessed whether we were able to replicate the bimodal adaptation findings of the main experiment in the control experiment. A comparison of the bimodal adaptation effects between the main and the control experiment using an ANOVA with experiment (main vs. control), motor and visual adaptors as factors revealed only an significant effect of visual adaptation, F(1, 35) = 132.56, p < 0.001, but no significant main effect or an interaction containing experiment as a factor, p > 0.05. Hence, the non-significant main effect of visual adaptation on hand movement speed is difficult to explain in terms of a general ineffectiveness of the visual adaptor.

Is the blocking of visual information during motor adaptation necessary for motor adaptation to occur? For example, the lack of visual information might have resulted in an effective spread of activation from the motor to the visual system in the unimodal but not bimodal condition. If simply the absence of visual information is required for motor adaptation to occur then opening the eyes during unimodal motor adaptation should decrease the motor adaptation effect. To test this hypothesis, participants were also tested in the unimodal motor adaptation condition with their eyes open looking at a blank screen. We examined the effect of eye state (open vs. closed) and adaptor (fist-bump vs. punch) on action categorization during motor adaptation with a two way within subjects ANOVA. The results showed a significant main effect of adaptor, F(1, 21) = 9.11, p = 0.007, no significant main effect of eye state, F(1, 21) = 1.58, p = 0.276, and no interaction between eye state and adaptor, F(1, 21) = 1.58, p = 0.222. Hence, the eye state (open vs. closed) seems to have little influence on motor adaptation.

## Discussion

We would like to point out that the variability of the motor and visual adaptation were likely to be associated with similar spatial variability. The average within subject variability of hand movement across all conditions was 3.2 cm (SD = 1.2 cm) confirming that participants executed the actions in a very consistent way. As for the variability of visual action information, we used head tracking to mimic natural viewing conditions and automatically updated the participants’ view of the three-dimensional avatar with the participants’ head movements. Although, we did not record the head movement data, other research suggests that head movement sway in the lateral medial plane while standing is about 2–3 cm^29^ and therefore of comparable magnitude to the motor movement variability. It therefore seems that despite comparable movement variability, action adaptation effects are still larger than motor adaptation effects.

What could be a reason for the weaker effect of the motor system on action recognition during motor-visual stimulation? One possibility is that motor processes involved in action recognition and motor processes involved in motor control compete for the same neural resources. One can speculate that processes dedicated to the primary purpose of the motor system to control limb movements prevail under conditions of simultaneous motor visual stimulation.

Do the action adaptation effects reported here occur at a perceptual level? An alternative explanation where action adaptation merely affects processes at a non-visual decision level predicts that adaptation effects are not or only to a small degree dependent on seeing bodily action information. Previous research provides results inconsistent with this view. Specifically, adaptation with action words (e.g. the word ‘hitting’) did not induce action adaptation aftereffects^21^. Hence, visual action information is important for the emergence of action adaptation effects. Moreover, the observation that adaptation effects depend on the attended visual features is inconsistent with the idea that adaptation effects only affect the decisional level. Specifically, de la Rosa and colleagues^30^ found different adaptation effects in an action discrimination (i.e. reporting whether the action was a handshake or a hig-five) and direction discrimination (i.e. reporting whether the stimulus moved forward or backward) task although the stimulus material in both tasks was identical. If the adaptor had an effect on the decisional level, all experimental conditions using the same adaptor should be associated with a very similar adaptation effects. However, we found the adaptation effect of the same adaptor to depend on whether participant attended to the type of action or its movement direction. Hence, the results suggest that adaptation effects depend on the visual features that participants are attending to (movement direction or action type). In sum, these results challenge the view that action adaptation effects are only based on non-visual decisional processes. Moreover, we provide a demonstration where the reader can visually experience the action adaptation effect (http://tinyurl.com/visadaptation).

The findings make an important contribution to a central debate in visual neuroscience regarding the effect of the motor system on visual action recognition. While previous research has mainly focused on investigating the degree to which the visual and motor modality contribute to action recognition, we examined the interaction of visual and motor action information. Specifically, we probed action recognition in situations of simultaneous action observation and execution. Our results strongly suggest that visual information dominates action recognition in these situations. Moreover, we found the influence of motor action information on action recognition to be significantly reduced in bimodal compared to unimodal conditions. Taken together these findings suggest that the effect of the motor system on action recognition is confined to situations, which do not require simultaneous action observation and action execution (e.g. pure action observation). Because simultaneous execution and observation of actions are key features of many social interactions (e.g. during the generation of a complementary fist bump), our results highlight the dominance of visual action processes for action recognition in social interactions. Hence, the effect of the motor system under more realistic viewing conditions, such as simultaneous observation and execution of actions, on social cognitive abilities requires further investigation.

Our results are in line with recent reports contrasting the visual and motor hypotheses of action recognition. For example, upper limb dysplasia (severe shortening of upper limbs), which is assumed to result in no motor representation for the corresponding limb, does not influence the ability to perceive, memorize, predict recognize, and anticipate upper limb actions (e.g. playing the guitar) compared to non-dysplasic controls^31^. This research suggests that action recognition is successful when it mainly relies on visual action information. The suggestion is in line with the results of our bimodal condition which indicate that action recognition mainly relies on visual information. At the same time, the lack of a motor effect in the above mentioned study does not preclude the possibility that the motor system affects action recognition in non-dysplasic patients. The results of the unimodal motor adaptation condition in the present study in fact suggests that the motor system has the ability to influence the visual categorization of an action. In a similar vein, it has been shown that cortical activation patterns during action observation depends on the abstraction level of the observed action. Specifically, the observation of actions from the same high abstraction level (e.g. viewing the opening of bottles or boxes ) is associated with activation in the lateral occipitotemporal cortex (LOTC) but not the ventral premotor cortex (PMv)^32^,^33^. Yet, the observation of actions of the same concrete level (e.g. viewing the opening of a specific box) is associated with activation in PMv. The result implies that the motor system might only be involved in the recognition of actions of a lower abstraction level. Our study provides behavioural evidence for causal relationship between motor adaptation and visual categorization of actions at concrete abstraction level (for categorization levels see^34^). In addition, we extend this finding by demonstrating that such effects might only occur during action observation but not during interaction.

It is important to note that the non-significant influence of motor action information on bimodal adaptation effects is not owed to the method’s lack of sensitivity to detect motor influences on action recognition. The results of the unimodal motor adaptation condition provides direct evidence for action execution influencing action discrimination in the absence of visual action information. To our knowledge this is the first evidence that shows that motor execution influences the discrimination of social actions given that no concurrent visual action information is present. Our results, thereby, partly support the long standing proposal^8^ that the motor system affects action recognition. Yet, future research needs to determine whether the influence of motor adaptation is mediated by a simulation mechanism as proposed by motor based approaches or by some other alternative mechanism, e.g. the activation of visual action representations by imagination of the action during motor execution.

In sum, visual action information dominates bimodal action adaptation effects suggesting that action recognition relies mainly on visual action information in situations of concurrent action observation and execution. However this motor visual influence might be subject to further modulation by e.g. spatio-temporal synchrony between motor and visual information. The results have implications for motor based social cognitive theories, which associate human social behaviour in social interactions with motor-based visual processes. Because simultaneous action observation and execution of actions is common in social interactions, our results suggest caution when associating social behaviour in social interactions with motor-based processes^9^,^35^,^36^,^37^.

## Methods

### Participants

22 participants from the local community in Tübingen participated in the experiment after giving written informed consent regarding their participation. The study was approved by the ethics review board of the University of Tübingen and all experiments were performed in accordance with relevant guidelines and regulations.

### Stimulus and apparatus

Stimuli were presented using an augmented reality setup consisting of a large screen display combined with a back-projection technique employing a Christie Mirage S + 3 K stereo projector (Kitchener, Canada) with a 101 Hz refresh rate. To see the stimuli in 3D, participants wore Nvidia 3D Vision Pro shutter glasses (Santa Clara, US) synchronized to the display. Head tracking with an ART Smarttrack (Weilheim, Germany) was used to update the visual scene in response to viewpoint changes. The punch and fist bump actions were motion captured real actions of humans using Xsens MVN suits (Enschede, Netherlands). The motion captured data was used to animate a life size female Rocketbox (Hannover, Germany) avatar (height 1.73 m) with a neutral facial expression, which directly faced the participant from 2.3 m away. Participants wore a marker on their right hand, which was used for action execution tracking and visual categorization responses (i.e categorizing the ambiguous stimulus as either ‘fist bump’ or ‘punch’). The hand movements were recorded using the Smarttrack system. Test stimuli were created by calculating the weighted average of local joint angles between the two actions. To determine weights for the ambiguous test actions, we presented a set of seven morphed actions to every participant and adjusted the weights until the participant reported overall ambiguous perception for all actions of the set. All stimuli had a length of 1.12 s.

### Procedure

At the very beginning of the experiment, participants saw each test stimulus three times in random order without any adaptation to measure action categorization in the absence of adaptation (baseline condition). The three repetitions of the seven test stimuli resulted in a total of 21 trials. In the actual experiment, there was an initial adaptation phase in which the same action information (punch or fist bump within the visual, motor, or combined visual and motor modality) was presented and/or executed 30 times with an inter-stimulus interval of 500 ms. For the first 29 presentations, each adaptor was accompanied by a presentation of a 1000 Hz tone that matched the length of the visual adaptor; the 30th and hence last adaptor was accompanied by a 1500 Hz tone. Subsequently, visual categorization of the test stimulus was probed once. Participant gave their answer (with their tracked hand) by touching one of two virtual square buttons hovering in mid-air in front of them which were labelled ‘punch’ and ‘fist bump’. Thereafter participants were re-adapted to the same action four times before performing a new categorization trial on a randomly chosen test stimulus. Specifically, each adaptation condition probed the perception of the seven test stimuli three times. Every participant participated in each of the eight adaptation conditions probing a different adaptor (unimodal: punch_vis_, punch_mot_, fist bump_vis_, fist bump_mot_; bimodal: punch_vis_/punch_mot_, fist bump_vis_/fist bump_mot_, punch_vis_/fist bump_mot_, fist bump_vis_/punch_mot_). The testing order of the adaptation conditions was randomized across participants. Only in the unimodal motor adaptation conditions, participants were told to execute the motor action while having their eyes closed and hearing a 1000 Hz tone. Participants were instructed to open their eyes at the end of the higher pitched 1500 Hz tone. During bimodal adaptation, the presentation of this sound was synchronized to the visual adaptor. For each adaptation condition we calculated the proportion of punch responses for the calculation of the adaptation effect.

## Additional Information

**How to cite this article**: de la Rosa, S. *et al.* Visual adaptation dominates bimodal visual-motor action adaptation. *Sci. Rep.* **6**, 23829; doi: 10.1038/srep23829 (2016).

## Figures and Tables

**Figure 1:**
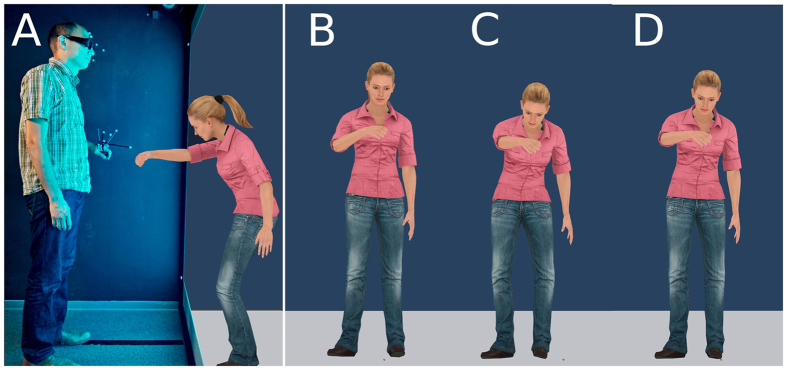
Schematic side view of the experimental setup. (**A**) Using shutter glasses participants had a 3D percept of a life-size avatar, who carried out the actions toward them. Reflective markers on the hand and glasses were used for tracking the hand and the head movement, respectively. Stimuli: (**B**) The peak frame of the dynamic fist bump adaptor. (**C**) the peak frame of the dynamic punch adaptor. Each adaptor was shown repeatedly. After adaptation, participants classified ambiguous test actions (one example shown in (**D**) Note, depicting the original dynamic stimuli as a static figure attenuates differences between tests and adaptor stimuli. The avatar is a Rocketbox asset.
